# Prospective assessment of cardiovascular risk parameters in patients with rheumatoid arthritis

**DOI:** 10.1186/s12947-018-0136-9

**Published:** 2018-09-18

**Authors:** Bożena Targońska-Stępniak, Mariusz Piotrowski, Robert Zwolak, Anna Drelich-Zbroja, Maria Majdan

**Affiliations:** 1 Department of Rheumatology and Connective Tissue Diseases, Medical University of Lublin, ul. Jaczewskiego 8, 20-950 Lublin, Poland; 2 Department of Interventional Radiology and Neuroradiology, Medical University of Lublin, ul. Jaczewskiego 8, 20-950 Lublin, Poland

**Keywords:** Rheumatoid arthritis, Carotid intima media thickness, Cardiovascular disease, Inflammation, Atherosclerosis

## Abstract

**Background:**

The study presents a prospective follow-up assessment of cardiovascular (CV) risk parameters in patients with rheumatoid arthritis (RA) in comparison with control subjects.

**Methods:**

The study group consisted of 41 RA patients. The following parameters were assessed at subsequent visits [initial (T0), follow-up after 6 years (T6)]: traditional CV risk factors, carotid intima media thickness (cIMT), QTc duration, serum concentration of amino-terminal pro-brain natriuretic peptide (NT-proBNP). A comparative cIMT assessment was performed on 23 healthy controls of comparable age.

**Results:**

The mean (SD) cIMT value in RA patients was significantly higher at T6 than at T0 [0.87 (0.21) vs 0.76 (0.15) mm, *p* < 0.001], the increase in patients with atherosclerotic plaques was noted. Patients with plaques were significantly older, had higher inflammatory parameters. The mean cIMT was significantly higher in RA patients than in controls at both T6, T0 visits. Certain traditional CV risk factors exacerbated during follow up. Unfavorable metabolic parameters and significantly higher cIMT were found in male patients than in female patients at T6. During follow-up, no significant differences in NT-proBNP, QTc were found. There were no significant relationships between cIMT, NT-proBNP, QTc and parameters of disease activity at T6.

**Conclusions:**

During the 6-year course of established RA, significant exacerbation of atherosclerosis was found, revealed by higher cIMT. A careful monitoring should be applied to patients with atherosclerotic plaques and of male gender due to higher burden of CV risk. In long-standing disease, traditional CV risk factors seem to play a key role, beyond the inflammatory activity.

## Background

Rheumatoid arthritis (RA) is a chronic, progressive, inflammatory joint disease, associated with increased risk of premature atherosclerosis and cardiovascular disease (CVD), to the similar extent as type 2 diabetes [1, 2]. Cardiovascular (CV) death is the leading cause of mortality of patients with RA, responsible for approximately 50% of deaths [3–5]. Traditional risk factors do not fully explain the increased CV risk. Chronic inflammation and high disease activity are reportedly associated with atherosclerotic burden, higher incidence of CVD, chronic heart failure (CHF), and mortality of patients with RA [6, 7].

The increased carotid intima-media thickness (cIMT) and presence of plaques are accepted as strong predictors of generalized atherosclerosis and major CV events in both non-RA and RA subjects [8, 9]. Higher cIMT was reported in RA patients compared with controls [8–11]. Significantly higher N-terminal pro-brain natriuretic peptide (NT-pro-BNP) levels were reported in RA patients, associated with RA duration, disease activity, and inflammatory markers, suggesting a link between inflammation and cardiac stress [11–14]. A relationship was observed between NT-proBNP and cIMT [13]. The increased QTc values were found in RA patients, associated with parameters of disease activity, severity and inflammation [4, 15].

The aim of the study was a prospective assessment of CV risk parameters in patients with established RA, in relation to disease activity and traditional CV risk factors.

## Methods

### Patients and controls

The study group consisted of RA patients treated at the Department of Rheumatology and Connective Tissue Diseases, Medical University of Lublin. All patients met the American College of Rheumatology (ACR)/European League Against Rheumatism (EULAR) classification criteria for RA [16]. The study was conducted in full compliance with the Helsinki Declaration. The protocol was approved by the Ethics Committee of Medical University of Lublin, with the approval number KE-0254/134/2013. The informed consent for participation in the study was obtained from all participants (patients and controls), after an adequate explanation of the study design.

The study was a part of a research program involving RA patients, to prospectively analyze CV risk factors, both traditional and non-traditional. The assessment presented in the study was performed twice at an average interval of 6 years [73.4 (6.4) months (59–87)], at baseline visit (T0) and current follow-up after 6 years (T6).

### RA-related data collection

Demographic and clinical data were obtained through structured interview, review of medical records, self-report questionnaires, and physical examination.

Disease activity was measured using the Disease Activity Score based on evaluation of 28 joints (DAS28), calculated with the number of tender, swollen joints, erythrocyte sedimentation rate (ESR), patient’s global disease activity assessment in visual analogue scale (VAS) [17].

The inflammatory burden within the 6-year course of RA was assessed as average of several results of C-reactive protein (CRP). Samples for CRP assessment were taken at consecutive visits, approximately every 6 months.

Erosive form of RA was diagnosed in patients with bone erosions in radiograms of hands and/or feet. Ability to perform daily activities was measured using modified Health Assessment Questionnaire (M-HAQ) [18].

### Laboratory tests

Blood was collected after overnight fasting to determine blood cell counts, ESR, serum concentration of CRP, creatinine (Cr), uric acid, glucose, total cholesterol (TC), high-density lipoprotein cholesterol (HDL-C), low-density lipoprotein cholesterol (LDL-C), triglycerides (TG) at the central laboratory of University Hospital. CRP was measured by immunoturbidimetric assay (upper limit 5 mg/l). Concentrations of TC, HDL-C, TG were measured with standard enzymatic technique (BIOMAXIMA); LDL-C was calculated according to Friedewald formula. Atherogenic index (AI) calculated as ratio TC/HDL-C seems to be more appropriate to assess CV risk in RA than individual cholesterol fractions (normal AI: <4.0 in women and <4.5 in men) [19].

Serum samples were stored at −80^o^ C for further assessment of NT-pro-BNP. Measurement of NT-proBNP concentration was performed using chemiluminescent immunometric assay (IMMULITE 2000 NT-proBNP, Siemens). Reference range according to manufacturer’s guidelines is up to 125 pg/ml in patients <75 years and up to 450 pg/ml in older; analytical sensitivity 10 pg/ml. BNP and NT-proBNP levels ≥100 pg/ml are significantly related to cardiac morbidity and mortality [12].

### Metabolic and CV biomarkers

Patients were classified as current, ex-smokers or non-smokers. Information about concomitant diseases was taken from medical records. Physical inactivity was defined as lack of regular training. Blood pressure (BP) was assessed in a sitting position. Height and weight were measured barefoot wearing light clothes. Body mass index (BMI) was calculated as the ratio of weight and squared height.

The 10-year risk of fatal CVD using Systemic Coronary Risk Evaluation (SCORE) model was estimated in every patient, with the value ≥5% indicating high or very high risk. According to the EULAR recommendations, the result was multiplied by 1.5 (mSCORE) [20].

### Carotid intima-media thickness (cIMT) measurement

Assessment of cIMT was performed at baseline (T0) and follow-up visit (T6) in 41 RA patients and 23 controls. All examinations were performed by the same experienced examiner, with the subject in a supine position, in a quiet, temperature-controlled room; using high-resolution B-mode ultrasound (US) (Logiq 7 GE). IMT was assessed bilaterally in three regions: common carotid artery (CCA), carotid bulb (BULB) and internal carotid artery (ICA). The analyses used the average of maximum IMT from 6 carotid segments (mean cIMT). The cIMT value <0.6 mm is considered as normal, ≥0.9 mm as abnormal. The cIMT value ≥0.6 mm and <0.9 mm is a marker of subclinical atherosclerosis [21]. Plaques were defined as a distinct protrusion >1.5 mm into vessel lumen, with their presence as marker of advanced atherosclerosis [22].

### Electrocardiogram assessment

The standard 12-lead transthoracic electrocardiogram (ECG) at 25 mm/s was performed for every patient. Measurement of QTc was performed automatically with normal value 350–430 ms for women and 350–450 ms for men, respectively [23].

### Statistics

Variables were tested for normality using the Kolmogorov-Smirnov test. Group differences were tested using Student’s t-test and Mann-Whitney U-test for normally and non-normally distributed parameters, respectively. Spearman’s or Pearson’s correlation test was used to determine association between clinical and laboratory variables. Multivariable analysis (multiple linear regression) was performed according to a forward selection procedure, introducing those variables that showed statistically significant association with certain parameters. For all tests, *P* values < 0.05 were considered significant.

## Results

### Demographic, CV risk and RA-related variables in patients

A clinical characteristics of RA patients at T6 has been presented in Table 1.

**Table 1:** Clinical characteristics of 41 RA patients at follow-up visit (T6)

Variables	Results
Demographic variables:	
Age, years	53.3 (8.7) (28–68)
Gender, F/M	34 (82.9)/ 7 (17)
Cardiovascular risk factors:	
Family history of CVD	20 (48.8)
Current/Ex-smokers	23 (56.1)
Hypertension	17 (41.5)
Diabetes	3 (7.3)
CKD3a (eGFR 45–59 ml/min/1.73 m^2^)	2 (4.9)
AI abnormal	11 (26.8)
BMI > 30 kg/m^2^	5 (12.2)
Physical inactivity	23 (56.1)
RA related variables	
Disease duration, years	19.2 (9.2) (8–45)
Erosions (X-ray of hands/feet)	35 (85.4)
Extra-articular symptoms	28 (68.3)
Positive RF-IgM	33 (80.5)
Positive anti-CCP2	30 (73.2)
ESR, mm/h	19.2 (16.0) (2–64)
CRP, mg/l	14.35 (37.5) (0.1–228.5)
Average CRP (6 years), mg/l	11.02 (9.9) (0.9–37.3)
Low RA activity (DAS28 ≤ 3.2)	23 (56.1)
Current glucocorticoid use	18 (43.9)
Current conventional synthetic DMARD	39 (95.1)
Current biological DMARD	32 (78.1)

Data are presented as mean (SD) (range) or number (%); *AI* atherogenic index, *Anti-CCP2* anti-cyclic citrullinated peptide antibodies, *BMI* body mass index, *CVD* cardiovascular disease, *CKD* chronic kidney disease, *CRP* C-reactive protein; *DAS28* disease activity score in 28 joints, *DMARD* disease modifying antirheumatic drug, *eGFR* estimated glomerular filtration rate, *ESR* erythrocyte sedimentation rate, *RF-IgM* IgM rheumatoid factor

The study group consisted of 41 patients with long-standing, advanced RA. The disease activity was low (DAS28 ≤ 3.2) in over 50% of patients. The value of average CRP (6 years) was slightly above normal range. Most patients had an erosive form of RA and were seropositive (RF-IgM and/or anti-CCP2). Extra-articular manifestations (rheumatoid nodules, sicca syndrome, interstitial lung disease, vasculitis) in the course of the disease were observed in almost 70% of patients (Table 1).

Traditional CV risk factors occurred frequently in RA patients (Table 1).

At T6, disease-modifying antirheumatic drugs (DMARDs) were not used in one patient. Conventional synthetic DMARDs (csDMARDs) used in 39 patients included: methotrexate (MTX) in 33 patients (80.5%), leflunomide 2 (4.9%), antimalarials (hydroxychloroquine or chloroquine) 12 (29.3%), cyclosporine 1 (2.4%). Biological DMARDs (bDMARDs) included: anti-TNF in 12 (29.3%), rituximab 14 (34.1%) and tocilizumab 6 (14.6%).

### Characteristics of the healthy volunteers group

The control group consisted of 23 healthy volunteers: 15 women (65.2%) and 8 men (34.8%), with the mean (SD) age of 49.6 (6.2) years (39–62), BMI 25.3 (3.1) kg/m^2^ (22.2–31.2). At T6, 10 (43.5%) controls were current/ex-smoker and 6 (26.1%) controls had arterial hypertension.

The mean age and BMI did not differ statistically between patients and controls.

### Comparison of cIMT between RA patients and controls

The mean value of cIMT was significantly higher in patients than in controls at both visits: current (T6) and baseline (T0) (Table 2).

**Table 2:** Comparison of cIMT and atherosclerotic plaques at T0 and T6 visits in the group of RA patients and controls

Parameters	Group	T0	*p*	T6	*p*
Mean cIMT, mm	RA patients	0.76 (0.15) (0.43–1.2)	< 0.001	0.87 (0.21) (0.57–1.77)	0.03
	Controls	0.59 (0.11) (0.4–0.87)		0.76 (0.11) (0.61–1.07)	
Abnormal cIMT (≥0.9 mm)	RA patients	9 (21.9)	0.6	14 (34.1)	0.6
	Controls	1 (4.3)		3 (13.0)	
Atherosclerotic plaques presence	RA patients	6 (14.6)	NS	10 (24.4)	NS
	Controls	1 (4.3)		4 (17.4)	
Bilateral atherosclerotic plaques presence	RA patients	3 (3.7)	NS	7 (17.1)	NS
	Controls	0		1 (4.3)	

Data are presented as mean (SD) (range), *cIMT* carotid intima media thickness

The significant increase of cIMT value during the 6-year follow-up was noted in both RA patients (*p* < 0.001) and controls (*p* < 0.001). The average increase of cIMT between T0 and T6 (delta IMT) was not significantly different between patients and controls [0.11 (0.18) vs 0.17 (0.09) mm, NS]. Carotid plaques were observed more often in patients than in controls at both T0 and T6 (statistically nonsignificant) (Table 2).

### Comparison of clinical and laboratory parameters at T0 and T6 in RA patients

Disease activity assessed with DAS28 diminished significantly between T0 and T6 (Table 3).

**Table 3:** Comparison of clinical and laboratory parameters at T0 and T6 in 41 RA patients

Variables	T0	T6	*p*
Clinical parameters			
DAS28	4.46 (1.15) (2.58–6.55)	3.33 (1.62) (0.66–6.88)	< 0.001
M-HAQ	1.22 (0.52) (0–2.38)	1.31 (0.52) (0.25–2.38)	NS
SBP, mmHg	123.8 (13.5) (90–160)	128.2 (13.6) (105–160)	NS
BMI, kg/m^2^	24.8 (3.1) (18.6–29.95)	25.9 (3.4) (17.3–33.2)	0.002
BMI > 30 kg/m^2^	0	5 (12.2)	0.03
mSCORE, %	0.98 (2.1) (0–12)	2.99 (3.9) (0–18)	< 0.001
High/very high CVD risk	2 (4.9)	10 (24.4)	0.01
QTc, ms	341.9 (60.8) (187–446)	358.8 (59.5) (175–453)	NS
cIMT, mm	0.76 (0.15) (0.43–1.2)	0.87 (0.21) (0.57–1.77)	< 0.001
Carotid plaques presence	6 (14.6)	10 (24.4)	NS
Laboratory parameters			
NT-proBNP, pg/ml	88.9 (78) (12.2–351.2)	126.6 (186.5) (20.1–1175)	NS
Glucose, mg/dl	89.3 (12.9) (54–120)	88.8 (14.5) (67–146)	NS
TC, mg/dl	200.6 (42) (135–325)	206.3 (7.2) (101–318)	NS
HDL-C, mg/dl	59.6 (14.2) (39–87)	60.3 (16.2) (30–102)	NS
LDL-C, mg/dl	119.7 (36.2) (47–232)	120.8 (34.4) (41–190)	NS
Triglycerides, mg/dl	106.5 (45.6) (43–214)	113.0 (53.5) (23–251)	NS
AI (TC/HDL-C)	3.48 (0.81) (2.1–5.5)	3.53 (0.84) (2.0–5.8)	NS
Serum creatinine, mg/dl	0.68 (0.15) (0.4–1.0)	0.66 (0.15) (0.4–1.0)	NS
Serum uric acid, mg/dl	4.0 (0.9) (2.2–5.8)	4.5 (1.1) (2.5–7)	< 0.05

Data are presented as mean (SD) (range) or number (%); *AI* atherogenic index, *BMI* body mass index, *cIMT* carotid intima media thickness, *DAS28* disease activity score in 28 joints, *HDL-C* high-density lipoprotein cholesterol, *LDL-C* low-density lipoprotein cholesterol, *M-HAQ* modified health assessment questionnaire, *NT-proBNP* amino-terminal pro-brain natriuretic peptide, *SBP* systolic blood pressure, *mSCORE* modified Systemic Coronary Risk Evaluation, *TC* total cholesterol

Significantly higher cIMT value was found in RA patients at T6 compared with T0 [0.87 (0.21) mm vs 0.76 (0.15), *p* < 0.001] (Table 3).

**Fig. 1:**
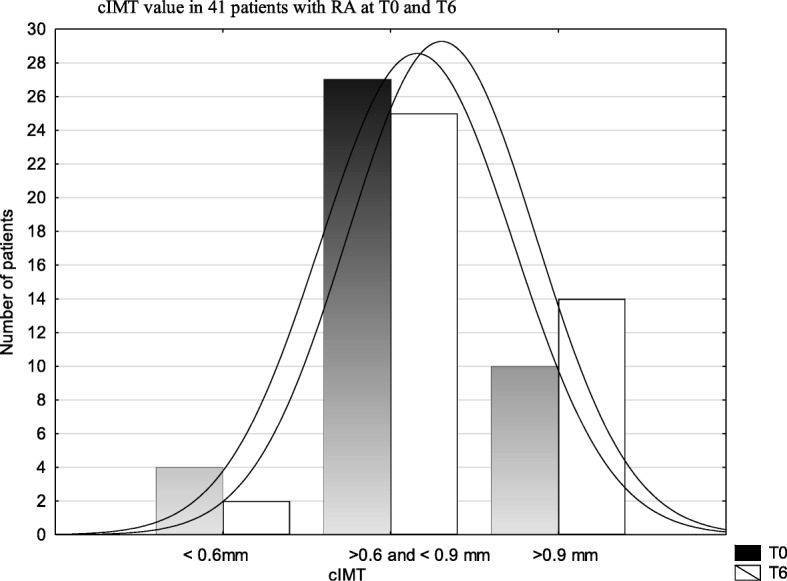
Comparison of cIMT value in 41 patients with RA at T0 and T6

**Fig. 2:**
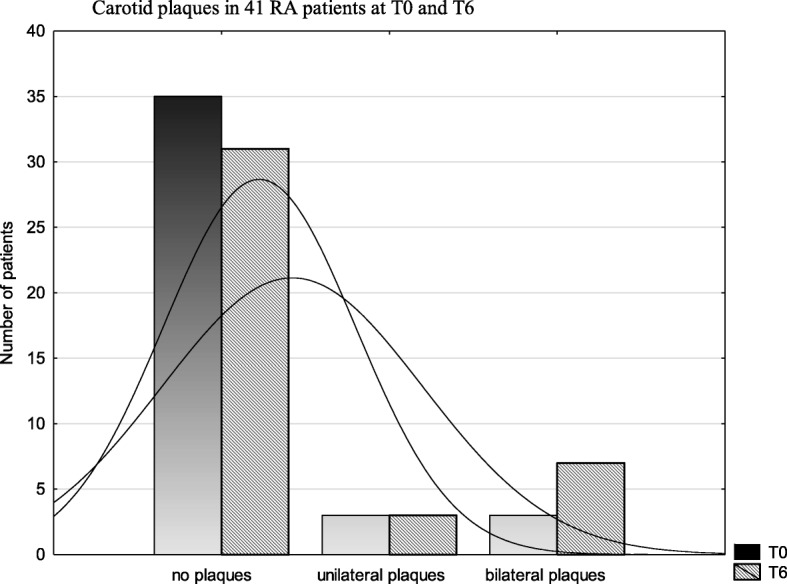
Comparison of atherosclerotic carotid plaques in 41 RA patients at T0 and T6

Most patients had increased cIMT value at both T6 and T0 assessments (Fig. 1). However, during the 6-year follow-up, an increase in the number of patients with defined atherosclerosis (cIMT ≥0,9 mm) (Fig. 1) and atherosclerotic plaques (Table 3) was observed (statistically nonsignificant). The number of patients with bilateral plaques increased between T0 and T6 (statistically nonsignificant) (Fig. 2).

Serum NT-proBNP concentration and QTc duration did not change significantly between T0 and T6 (Table 3).

Certain traditional CV risk factors (serum uric acid, BMI) and mSCORE exacerbated between T0 and T6 (Table 3). The number of patients with high/very high risk of CV death increased significantly (Table 3).

The other clinical and laboratory parameters did not change significantly (Table 3).

### Relationship between CV risk parameters at T0 and T6 in RA patients

There was a strong relationship between the mean cIMT values assessed in RA patients at T6 and T0 (*p* < 0.001). In multiple linear regression analysis, significant association at T0 was confirmed between cIMT and SCORE (*p* = 0.001), NT-proBNP (*p* = 0.04). This relationship was not observed at T6. There were no significant relationships between cIMT, NT-proBNP, OTc and parameters of current disease activity or inflammation as well as average CRP.

### Comparison of patients with and without atherosclerotic plaques

Patients with atherosclerotic plaques at T6 were older [59.0 (5.7) vs 51.5 (8.7), *p* = 0.02], had significantly higher CRP concentration [40.6 (72) vs 6.2 (9.5), *p* = 0.01] and white blood cell count (WBC) [7.6 (2.6) vs 5.5 (1.6), *p* = 0.006].

### Comparison of male and female RA patient

At T0, the mean cIMT value was non-significantly higher in men than in women with RA [0.82 (0.2) vs 0.75 (0.14), NS]. However, at T6, unfavorable CV and metabolic parameters were found in male compared with female patients: significantly higher cIMT [1.1 (0.33) vs 0.82 (0.14), *p* < 0.001], SCORE [6.0 (6.1) vs 2.3 (2.9), *p* = 0.04], uric acid [5.5 (1.0) vs 4.3 (1.0), *p* = 0.007] and lower HDL-C [44.3 (10.7) vs 63.1 (15.4), *p* = 0.007].

In control subjects, the mean cIMT at T6 did not differ significantly between men and women [0.77 (0.13) vs 0.76 (0.1), NS].

## Discussion

In this prospective, 6-year follow-up study, exacerbation of atherosclerosis was observed in patients with advanced RA, revealed by a significant increase of cIMT value and an increase in patients with atherosclerotic plaques (non-significant). Patients with atherosclerotic plaques showed significantly higher inflammatory parameters than patients without plaques, suggesting association between inflammatory burden and plaques presence. Especially the presence of bilateral plaques is noteworthy due to significantly higher CV risk and the quadrupled risk of acute myocardial syndrome [24].

Exacerbation of atherosclerosis occurred despite effective control of RA activity. Disease activity according to DAS28 decreased significantly during follow-up, which was associated with active DMARDs treatment. Simultaneously deterioration was found in certain traditional CV risk factors. During follow-up the risk of CV death increased significantly and 25% of patients had high/very high risk of fatal CVD.

The current cIMT was significantly associated only with baseline cIMT assessed 6 years before. No significant relationship was found between current cIMT, NT-proBNP, QTc values and disease activity parameters. The results suggest, that initial state of CV system had a profound effect on further CV risk in the course of RA.

Exacerbation of atherosclerosis, revealed by higher cIMT, was also found in the control group. The cIMT increases were comparable in both groups (patients and controls), which suggests an effect of physiological aging. Similar results were presented in a follow-up study, in which, after 5-year observation, significantly higher cIMT was found in both early RA patients and controls, atherosclerotic plaques were not mentioned [11].

Atherosclerotic plaques were shown to be more predictive of CV events than elevated cIMT, since cIMT value may be associated with arterial wall aging, body size, muscularity [25]. New or progressive carotid plaques were strongly associated with markers of inflammation and disease activity (CRP, swollen joints count) [25]. It seems that in RA, an additional, maladaptive arterial wall remodeling increases risk of plaque rapture which could explain highly increased CV risk despite a seemingly normal cIMT [26]. According to Semb et al., patients with RA had numerically more atherosclerotic plaques than controls, associated with the presence of RA, but not with the level of RA activity. However, patients in remission and controls had more stable plaques than patients with active disease, pointing to importance of achieving remission [27].

Several hypotheses have been proposed to explain the accelerated atherosclerosis in RA. Thus far, there is no evidence that RA-associated single-nucleotide polymorphisms (SNP) as a group are associated with coronary artery disease [28].

The greater burden of traditional CV risk factors is suggested among RA patients [28, 29]. It seems that classical CV risk factors may be important for generation and progression of stable atherosclerosis, whereas local and systemic high-grade inflammation may contribute to plaque instability and higher rates of acute CV syndromes [30]. Dalbeni et al. found that only age was consistently associated with cIMT and atherosclerotic plaques as major determinant of subclinical atherosclerosis [31]. Male gender is reported to have an impact on cIMT in addition to disease activity parameters [8]. According to data in literature, on average cIMT is higher in men than in women and increases with age [32].

In this study, cIMT value, which was comparable in male and female patients at initial (T0) assessment, became significantly higher in males at the 6-year follow-up. Simultaneously, a higher burden of traditional CV factors and risk of CV death was found in men with RA.

Systemic, immune-mediated inflammation significantly contributes to accelerated atherosclerosis [6]. Effective control of inflammation as a result of DMARDs treatment is associated with CV risk reduction. Significantly lower cIMT was reported in RA patients treated with MTX and correlated with MTX dosage [33]. Significant cIMT reduction was reported in RA patients treated with TNF inhibitors and steadily responsive to therapy [34].

In established RA, NT-proBNP was associated with the disease activity, duration, as well as cIMT [2, 5, 12, 13]. It was suggested that increased NT-proBNP could reflect silent myocardial stress associated with transient ischemia due to atherosclerosis [13]. It was reported that in RA patients without evident CHF, treatment with TNF inhibitor decreased NT-proBNP, suggesting the link between inflammation and cardiac stress [35]. In this study, non-significant increase of NT-proBNP was observed during follow-up, with no clinical symptoms of CHF. The significant association between NT-proBNP and cIMT at T0 was not found at follow-up, which may be considered as a result of active DMARDs treatment.

It seems that traditional CV risk factors in association with high-grade inflammation are responsible for acceleration of atherosclerosis in early course of RA. The results of our study indicate that at follow-up the burden of atherosclerosis is still higher in RA patients than in healthy controls and constantly related to the disease activity. Traditional CV risk factors seem to be important in long-standing advanced RA.

The careful diagnosis and management of CV risk factors should be considered for all RA patients, especially those with atherosclerotic plaques should be treated as high-risk patients.

The main strength of our present study is its prospective design. The same two groups of patients and controls were assessed with a 6-year interval. The detailed characteristics of patients considered all aspects of RA pathology. Another strength is the same specialist who performed all US measurements, thereby eliminating any interpersonal variations.

There are some limitations of the study, including quite small groups of patients and controls. The score used in the study (DAS28) presents only the current disease activity and does not reflect the activity burden over the 6 years. Therefore, further evaluation should be performed on a larger group of patients, considering assessment of the activity burden with other methods.

## Conclusions

In this prospective, 6-year follow-up study of patients with established RA, significant exacerbation of atherosclerosis was found, as revealed by higher cIMT and increase in patients with atherosclerotic plaques, despite significant reduction of disease activity. Unfavorable metabolic parameters and significantly higher cIMT in male compared with female patients were noted. The mean cIMT was significantly higher in RA patients than in controls at both assessments. Tight control of inflammation could be an effective method to reduce CV risk. However, in long-standing disease traditional CV risk factors seem to play a key role, beyond inflammatory activity of the disease.

## Abbreviations

ACR: American College of Rheumatology
AI: Atherogenic index
bDMARD: biological DMARD
BMI: Body mass index
BP: Blood pressure
BULB: Carotid bulb
CCA: Common carotid artery
CHF: Congestive heart failure
cIMT: carotid intima-media thickness
Cr: Creatinine
CRP: C-reactive protein
csDMARD: conventional synthetic DMARD
CV: Cardiovascular
CVD: Cardiovascular disease
DAS28: Disease Activity Score evaluated in 28 joints
DMARD: Disease-modifying antirheumatic drug
ECG: Electrocardiogram
ESR: Erythrocyte sedimentation rate
EULAR: European League Against Rheumatism
HDL-C: High-density lipoprotein cholesterol
ICA: Internal carotid artery
LDL-C: Low-density lipoprotein cholesterol
M-HAQ: Modified Health Assessment Questionnaire
NT-proBNP: N-terminal pro-brain natriuretic peptide
QTc: Heart rate-corrected QT interval
RA: Rheumatoid arthritis
SCORE: Systemic Coronary Risk Evaluation
TC: Total cholesterol
TG: Triglycerides
TNF: Tumor necrosis factor
US: Ultrasound
VAS: Visual analogue scale
